# Lactulose-Induced Ischemic Colitis: A Rare Presentation and an Overview of Possible Etiologies of the Disease

**DOI:** 10.7759/cureus.23774

**Published:** 2022-04-03

**Authors:** Zaryab Umar, Usman Ilyas, Deesha Shah, Nso Nso, Allison Foster, Milana Zirkiyeva

**Affiliations:** 1 Internal Medicine, Icahn School of Medicine at Mount Sinai, Queens Hospital Center, New York, USA; 2 Medicine, Ichan School of Medicine at Mount Sinai, NYC Health and Hospitals (H+H) / Queens, New York, USA

**Keywords:** spontaneous bacterial peritonitis, severe hepatic encephalopathy and chronic liver disease, pneumatosis intestinalis, live cirrhosis, ischemic colitis, lactulose

## Abstract

Ischemic colitis is one of the most common ischemic pathologies of the gastrointestinal system and can be divided into non-gangrenous and gangrenous forms. The pathophysiology involves restricted blood supply to the colonic mucosa. Several risk factors have been implicated in the development of ischemic colitis. Lactulose, one of the mainstay therapies for the treatment of hepatic encephalopathy in patients with cirrhosis, has been rarely reported as a cause of ischemic colitis. To the best of our knowledge, there has been only one case report associating lactulose use with the development of ischemic colitis. The exact pathophysiology is unknown but might be associated with the fermentation of lactulose by intestinal bacteria, causing gaseous distention and increasing the intraluminal pressure. We present the case of a 77-year-old African American male, a known case of non-alcoholic liver cirrhosis with portal hypertension and esophageal varices, brought in by his family to the emergency department for altered mental status, non-bilious vomiting, abdominal distension, and pain for one day. On physical examination, the patient had upper extremity asterixis and was alert but disoriented to place and person. Diagnostic paracentesis was performed, which revealed leukocytosis, predominantly neutrophils. The patient was admitted for spontaneous bacterial peritonitis and hepatic encephalopathy with decompensated liver cirrhosis. The patient was started lactulose with a goal of three to four bowel movements per day. Despite adequate treatment, the patient continued to develop worsening mental function and abdominal distension. This was later followed by a bloody bowel movement. Laboratory assessment showed an elevated white blood cell count, worsening kidney function, and high anion gap metabolic acidosis. CT scan revealed dilated loops of bowel with air and fluid along with submucosal wall edema, findings suggestive of ischemic colitis. Given the poor prognosis and the patient's condition, colonoscopy was deferred. Lactulose was discontinued, as it was thought to be a contributing cause of the patient's ischemic colitis. His condition continued to deteriorate, and he passed away on Day 18 of admission.

## Introduction

Ischemic colitis is one of the most common ischemic pathologies of the gastrointestinal system [1]. The likely pathophysiology is colonic arterial tortuosity and small vessel narrowing that potentially limit the blood supply to the colonic mucosa [2]. It is categorized into non-gangrenous and gangrenous types. Approximately 80%-85% of ischemic colitis cases correspond to the non-gangrenous form; however, 20%-25% reportedly account for chronic segmental colitis [3]. Ischemic colitis often remains underdiagnosed due to its non-specific clinical presentation that predominantly includes diarrhea, crampy abdominal pain, defecation urge, and maroon/bright red blood per rectum (or lower gastrointestinal bleeding) [4-6]. The recent findings indicate a 4.2% mortality rate within 30 days of diagnostic confirmation [4]. However, the inpatient survival rate in ischemic colitis cases ranges between 82%-85% within a span of 30-90 days [7]. An abdominal radiograph effectively rules out other acute intra-abdominal pathologies in patients with lactulose-induced ischemic colitis [8]. The diagnostic affirmation of ischemic colitis; however, depends on computed tomography (CT) and magnetic resonance (MR) imaging modalities that effectively track portal venous gas, pneumatosis, bowel dilation, colonic wall thickening, edema, and thumbprinting [9]. In addition, colonoscopy is the gold standard for diagnosing ischemic colitis based on its capacity to visualize colonic mucosa and facilitate histological sampling [10]. The CT findings for non-gangrenous ischemic colitis include pericolonic stranding, thumbprinting, and bowel wall thickening; however, the medical management relies on nasogastric tube placement, bowel rest, supplemental oxygen, cardiac output optimization, and intravenous fluid resuscitation. The management of the gangrenous form relies on surgical resection guided by Indium-111 labeled leukocyte scans [11]. The predisposing factors for ischemic colitis include trauma, thrombosis, mesenteric artery emboli, mechanical obstruction, colonic hypoperfusion, hypovolemia, and mechanical obstruction of the colon. The pharmacologic agents that trigger colonic ischemia include vasopressors, Non-steroidal anti-inflammatory drugs (NSAIDs), laxatives, immunosuppressive drugs, decongestants, chemotherapeutic agents, appetite suppressants, and antibiotics [3]. Lactulose-induced changes in the gut microenvironment also potentiate colonic hypoperfusion and subsequent colonic ischemia [12]. This case study elaborates on the clinical presentation of an elderly patient suspected of lactulose-induced ischemic colitis.

## Case presentation

We are presenting a case of a 77-year-old African American male who was brought in with his family to the emergency room with altered mental status, abdominal distention, abdominal pain, and non-bilious non-bloody vomiting for one day. The patient had a known history of non-alcoholic liver cirrhosis complicated by portal hypertension, and esophageal varices (status post esophageal banding done two months). Other comorbidities included obesity, advanced cataract bilaterally, advanced glaucoma, hypertension, hyperlipidemia, Heliobacter (H.) pylori-associated active gastritis, and anemia of chronic disease.

In the emergency department, the patient had a blood pressure of 164/84 mmHg, pulse of 84 beats per minute, a temperature of 98 °F, and oxygen saturation of 100% on room air. Physical examination revealed scleral icterus, abdominal distention, abdominal tenderness on palpation, tympanic abdomen with fluid thrill on percussion, bilateral +3 pitting edema of lower extremities, asterixis of the upper extremity, and alert but disoriented to place and person. With the high suspicion of ascites on physical examination, diagnostic paracentesis was performed in the emergency department and approximately 150 mL of amber color fluid was drawn. Initial laboratory assessment showed no leukocytosis (white cell count of 6.95 x10(3)/mcL), acute kidney injury with a creatinine of 1.56 mg/dL (the patient's baseline creatinine was 1.05 mg/dL), reduced glomerular filtration rate of 42, lactic acidosis with venous lactate of 4.8 mmol/L, transaminitis with aspartate aminotransferase (AST)/alanine transaminase (ALT) >2, elevated alkaline phosphatase (144 U/L), hyperbilirubinemia (total bilirubin of 3.5 with direct bilirubin of 1.7 mg/dL), worsening coagulopathy with international normalized ratio (INR) of 1.8, prothrombin time of 21.1, and activated partial thromboplastin time (aPTT) of 38.3 seconds (Table 1). The patient's urine analysis did not reveal any signs of infection. The patient had a Maddrey discrimination function of 47.7 points (poor prognosis) and a model for end-stage liver disease (MELD)-sodium score of 23 points (7%-10% estimated 90-day mortality). An ultrasound of the abdomen showed coarsened echotexture of the liver with nodularity suggestive of suspected liver cirrhosis with perihepatic ascites and cholelithiasis with no evidence of acute calculus cholecystitis, intrahepatic, and common bile ductal dilatation. CT abdomen without contrast showed similar findings suggestive of liver cirrhosis, cholelithiasis, and moderate abdominal as well as pelvic ascites. The ascitic fluid examination was significant for turbid appearance, leukocytosis to 7245/mcL with 86% neutrophils, with fluid gram stain and culture being negative. The patient had a quick sepsis-related organ failure assessment (qSOFA) score of 1. 

**Table 1:** Pertinent lab values on the day of admission and at the time of development of ischemic colitis aPTT: activated partial thromboplastin time; INR: international normalized ratio; BUN: blood urea nitrogen; ALT: alanine transaminase; AST: aspartate aminotransferase

Lab results (units and reference range)	Day of admission	Day 3 of admission/development of Ischemic colitis
Hemoglobin (14.0-18.0 g/dL)	13.1	10.9
White blood cell count (4.80-11.80 x10(3)/mcL)	6.95	8.44
Platelets (150-450 x10(3)/mcL)	169	50
aPTT (25.1-36.5 seconds)	38.3	63.8
INR	1.6	4.8
Lactic acid (0.5-2.2 mmol/L)	4.8	11.2
BUN ( 6-23 mg/dL)	19	42
Creatinine (0.70-1.20 mg/dL)	1.56	3.28
Sodium (136-145 mmol/L)	130	153
Potassium (3.5-4.1 mmol/L)	Hemolyzed	3.3
Glomerular filtration rate (ml/min)	42	17
Albumin (3.5-5.2 g/dL)	2.6	3.0
Total protein (6.6-8.7 g/dL)	8.1	5.4
Total bilirubin (0.0-1.20 mg/dL)	3.5	4.8
Direct bilirubin (0.0-0.3 mg/dL)	1.7	2.7
Alkaline phosphatase (40-129 U/L)	144	68
ALT (0-41 U/L)	53	33
AST (5-40 U/L)	299	93

The patient was admitted with the impression of spontaneous bacterial peritonitis with hepatic encephalopathy or decompensated liver cirrhosis. The patient had grade 2/overt hepatic encephalopathy on West Haven Criteria (WHC) and a Child-Pugh score of 13 (class C). He was started on cefotaxime 2 g every eight hours for spontaneous bacterial peritonitis (SBP) coverage, furosemide 40 mg daily, and spironolactone 50 mg daily. In addition, the patient was started on lactulose enema with a goal of three to four bowel movements per day.

**Figure 1:**
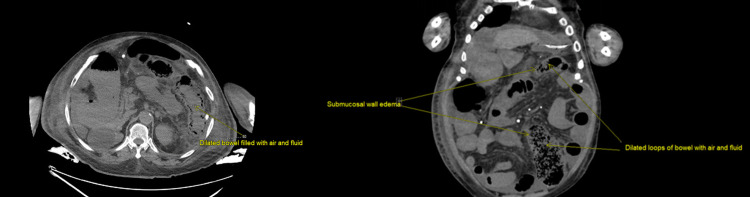
CT scan of the abdomen and pelvis revealing dilated loops of bowel with air and fluid along with submucosal wall edema, findings suggestive of ischemic colitis

On Day 2 of admission, therapeutic paracentesis was performed with the removal of 4 liters of ascitic fluid and was given 25% 25 g albumin following large-volume paracentesis. On the following day, despite receiving treatment and bowel movements of three to four per day, the patient had worsening mental status and worsening abdominal distention. The next day, the patient had a large bloody bowel movement with bright red blood per rectum. Vitals at that time showed mild hypothermia of 94.6 F and hypotension of 98/55 mmHg, which improved to 122/60 mmHg upon fluid resuscitation. Laboratory work showed normal leukocytes count with bandemia of 12, high anion gap metabolic acidosis, and base deficit with worsening lactic acid level of 11.2 (Table 1). Blood cultures drawn did not reveal any growth. Initial abdominal X-ray showed dilated small bowel loops with air in the colon. CT abdomen pelvis showed diffuse wall thickening in the transverse colon and descending colon with interval development of pneumatosis intestinalis. The clinical, laboratory, and imaging findings were suggestive of likely ischemic colitis (Figure 1).

At the same time, a family conference was held to discuss the prognosis and further care of the patient. The family decided to make the patient do not resuscitate/do not intubate (DNR/DNI) with no aggressive surgical/medical interventions. Even though endoscopy/biopsy of the colon is considered the gold standard in the diagnosis of ischemic colitis, considering the poor prognosis, high mortality, and respecting the wishes of the patient’s family, the decision was made to defer any aggressive interventions, including colonoscopy/biopsy. Given that the patient did not undergo any recent surgical intervention, had no history of cardiovascular disease, had negative blood cultures and a mean arterial pressure above 65 mmHg at the onset of bloody bowel movements, lactulose was thought to be the likely cause of the patient's ischemic colitis and hence was discontinued. The patient was started on intravenous fluid hydration, supplemental oxygen was given as needed, A nasogastric tube was inserted, bowel rest was provided, and broad-spectrum antibiotics ciprofloxacin and metronidazole were given as non-operative management. Despite continuing supportive treatment, intravenous fluid resuscitation, and broad-spectrum antibiotics, the patient's clinical condition deteriorated over time with worsening liver and renal functions and the patient passed away on Day 18 of admission.

## Discussion

Ischemic colitis is the inflammation of the gastrointestinal tract triggered by inappropriate vascular supply to the colon. The incidence and severity of ischemic colitis reciprocate with age advancement [13]. The diagnostic assessment of ischemic colitis in elderly patients is based on ruling out significant predictors, including vasculopathy, heart disease, cancer, and kidney dysfunction [14]. The conservative management of transient ischemic colitis depends on the degree of ischemic deterioration and helps improve healing and mucosal regeneration [15]. A mortality rate of 6.2%-39.3% is recorded based on clinical and surgical complications [2]. Elderly patients with ischemic colitis experience a mortality rate of 29% based on higher lactate levels and comorbidities [16]. The postoperative mortality after emergency open colectomy in ischemic colitis cases is attributed to postoperative acute kidney injury, delay in surgery, and elevated levels of preoperative lactates [17]. However, ischemic colitis in the treated patient proved fatal despite appropriate diagnostic management. The assessment of possible etiologies of ischemic colitis in elderly patients is therefore imperative to reduce the risk of preventable mortality. This case study delineates potential diagnostic limitations, including colonoscopy and colonic mucosal biopsy that help determine the etiology of ischemic colitis [15]. The other diagnostic methods include barium enema, CT angiography, and contrast-enhanced magnetic resonance angiography (MRA); however, their use is restricted due to potential limitations in the setting of ischemic colitis [18-19]. The underlying comorbidities and recurrent bleeding in severe ischemic colitis challenge its diagnostic management. A comprehensive assessment of etiological factors is, therefore, necessary to improve future clinical decision-making [20]. 

The contemporary literature reveals possible etiologies of ischemic colitis, including constipation-inducing drugs, hypoalbuminemia, diabetes mellitus, hypertension, hemodialysis status, and old age [21]. The assessment of these conditions in patients with lower abdominal discomfort is paramount to ruling out ischemic colitis. Patients with a history of vasculitis experience a high risk of ischemic colitis due to a reduction in intestinal blood flow and subsequent elevation in intraluminal pressure. The other possible causes include chronic constipation, acute pancreatitis, allergy, amyloidosis, cardiac arrhythmias, coagulopathies, infections, atheroembolism, volvulus, left atrial myoma, and penetrating trauma [22]. A cohort study by Suh et al. revealed a 2.78-3.17 times higher risk of ischemic colitis in patients with constipation and irritable bowel syndrome [23]. Young patients with cocaine-induced ischemic colitis experience a mortality rate of 26% [24]; however, methamphetamine-induced ischemic colitis manifests with necrotic mucosa and grade-II ischemia [25]. Antipsychotics, including haloperidol, cyamemazine, levomepromazine, and clozapine, add to a 55.2% risk of ischemic colitis [26]. Other prescription medicines that potentiate the incidence of ischemic colitis include antimuscarinic drugs, digoxin, and aspirin. In addition, type 1 interferons and tumor necrosis factor-alpha also add to the incidence of ischemic colitis [27]. Thrombophilia due to coagulopathy further increases the 28%-74% risk of ischemic colitis [15]. Recent research reveals the role of abdominal aortic aneurysm surgery in aggravating ischemic colitis and increasing its mortality rate [28]. The conditions, including paroxysmal nocturnal hemoglobinuria, protein S/C deficiency, polycythemia vera, and anti-thrombin deficiency, also increase the risk and incidence of ischemic colitis.

The disturbance in the anticoagulant and procoagulant reactions in liver cirrhosis triggers clot formation or excessive bleeding [29]. The portal hypertensive colopathy manifests with rectal varices, colonic vascular ectasias, non-specific inflammatory changes, and hemorrhoids [30-31]. The other manifestations include mild chronic inflammatory infiltrates, edema of lamina propria, and irregular thickening in dilated tortuous mucosal capillaries [32]. A retrospective study by Then et al. revealed a higher mortality rate (i.e.,10.7%), increased total hospital charge (i.e., USD 84,769), and longer length of stay (i.e.,7.3 days) in patients with ischemic colitis and history of cirrhosis [29].

In rare scenarios, ischemic colitis possibly develops due to gaseous distension triggered by lactulose fermentation via colonic bacteria [33]. The lactulose-induced intestinal ischemia probably develops after a decline in intestinal blood flow due to intraluminal pressure elevation. To the best of our knowledge, the case study by Anand et al. is the only evidence that clinically correlates ischemic colitis with lactulose therapy and is the only laxative mentioned in the literature that might contribute to the development of the disease [33].

The imaging results in the present case suggested ischemic colitis manifestations that probably developed after lactulose administration. In addition, portal hypertension and low albumin levels triggered by decompensated liver cirrhosis could have worsened the prognostic outcomes and attributed to the reported mortality [34]. These findings advocate the need for meticulously monitoring lactulose therapy in patients suspected of ischemic colitis.

## Conclusions

This case report unravels possible etiologies and causative factors of ischemic colitis. The writers do acknowledge other risk factors that might have contributed to the patient's disease such as old age, liver cirrhosis, and coagulopathy in the setting of liver disease. Given that our patient did not undergo any surgical intervention in the recent past, had no history of cardiovascular disease, had negative blood cultures, and had a mean arterial pressure of more than 65 mmHg at the time of onset of bloody bowel movements; lactulose was considered to be a possible etiology of the patient's ischemic colitis. It is our hope that the readers will consider lactulose in their differentials as the possible cause of ischemic colitis in patients with decompensated liver cirrhosis and hepatic encephalopathy who have been started on lactulose therapy. Prompt discontinuation might lead to better patient outcomes in such cases. Further research needs to be conducted in this area.
